# Prevalence and Associations of Type 2 Diabetes Risk and Sociodemographic Factors in Saudi Arabia: A Web-Based Cross-Sectional Survey Study

**DOI:** 10.3390/ijerph20032269

**Published:** 2023-01-27

**Authors:** Reem F. Bamogaddam, Yahya Mohzari, Fahad M. Aldosari, Ahmed A. Alrashed, Abdulaziz S. Almulhim, Sawsan Kurdi, Munirah H. Alohaydib, Ohoud M. Alotaibi, Amani Z. Alotaibi, Ahmad Alamer

**Affiliations:** 1 Department of Clinical Pharmacy, King Saud Medical City, Riyadh 12746, Saudi Arabia; 2 Department of Pharmacy, King Fahad Medical City, Riyadh 11564, Saudi Arabia; 3 Department of Pharmacy Practice, College of Clinical Pharmacy, King Faisal University, Al-Ahsa 31982, Saudi Arabia; 4 Department of Pharmacy Practice, College of Clinical Pharmacy, Imam Abdulrahman Bin Faisal University, Dammam 34221, Saudi Arabia; 5 Department of Pharmaceutical Services, King Saud Medical City, Riyadh 12746, Saudi Arabia; 6 Department of Clinical Pharmacy, Prince Sattam Bin Abdulaziz University, Alkharj 16273, Saudi Arabia

**Keywords:** prevalence, diabetes mellitus (DM), prediabetes, Canadian risk (CANRISK), screening, Saudi Arabia

## Abstract

Type 2 diabetes mellitus (T2DM) is a chronic disease with ever-increasing prevalence worldwide. In our study, we evaluated the prevalence of the risk of developing T2DM in Saudi Arabia and investigated associations between that risk and various sociodemographic characteristics. To those ends, a web-based cross-sectional survey of Saudi nationals without diabetes, all enrolled using snowball sampling, was conducted from January 2021 to January 2022. The risk of developing T2DM was evaluated using a validated risk assessment questionnaire (ARABRISK), and associations of high ARABRISK scores and sociodemographic variables were explored in multivariable logistic regression modeling. Of the 4559 participants, 88.1% were 18 to 39 years old, and 67.2% held a college or university degree. High ARABRISK scores were observed in 7.5% of the sample. Residing in a midsize city versus a large city was associated with a lower ARABRISK risk score (*p* = 0.007), as were having private instead of governmental insurance (*p* = 0.005), and being unemployed versus employed (*p* < 0.001). By contrast, being married (*p* < 0.001), divorced or widowed (*p* < 0.001), and/or retired (*p* < 0.001) were each associated with a higher ARABRISK score. A large representative study is needed to calculate the risk of T2DM among Saudi nationals.

## 1. Introduction

According to the International Diabetes Federation, in 2021, approximately 537 million people worldwide lived with diabetes, a number that is expected to reach 700 million by 2045 [1]. In Saudi Arabia, the prevalence of diabetes, at more than 25% of all adults, ranks among the highest in the world. According to the World Health Organization, Saudi Arabia ranks second in the prevalence of diabetes in the Middle East and seventh globally [2].

The likelihood of developing type 2 diabetes mellitus (T2DM) depends on a combination of modifiable and non-modifiable risk factors, as follows: first-degree family history of T2DM, high-risk race or ethnicity, overweight or obesity, sedentary lifestyle, being at least 35 years old, history of impaired glucose tolerance, impaired fasting glucose, elevated hemoglobin A1C, hypertension, dyslipidemia, history of cardiovascular disease, polycystic ovary syndrome, and other conditions associated with insulin resistance [3]. Recently, given the increased prevalence of T2DM, the American Diabetes Association (ADA) lowered the age threshold for T2DM screening in asymptomatic adults from 45 to 35 years [3], which also aligns with the Saudi National Diabetes Center’s (SNDC) recommendations for T2DM screening [4].

Many risk scoring models for T2DM require the findings of blood tests which, due to necessitating a clinic visit or diagnostic testing, complicates the wide use of those models from a public health perspective [5]. A method of evaluating the risk of diabetes based on self-report, however, eliminates the need for laboratory testing and may be cost-effective, reliable, valuable, and easy to use. Moreover, the widespread use of such an assessment tool could improve the public’s knowledge about the risk factors for diabetes, which has been shown to be lacking [6]. Beyond that, to determine whether a diagnostic test is relevant, the ADA advises practitioners to screen asymptomatic adults for the risk of prediabetes and T2DM using an informal assessment of risk factors for diabetes or an assessment tool [3]. To date, such tools include the Finnish Diabetes Risk Score (FINDRISC) [7], the Canadian Diabetes Risk Questionnaire (CANRISK) [8], the ADA’s tool [9], and the Cambridge Diabetes Risk Score [10]. Among these, FINDRISC provided the basis for CANRISK, which was modified to account for different ethnicities in Canada and has since been translated into Arabic as the Arab Diabetes Risk Assessment Questionnaire (ARABRISK). A tool validated by researchers in Saudi Arabia and Jordan [11], ARABRISK consists of questions addressing 12 risk factors for diabetes and categorizes individuals as being at very high risk, high risk, or low to moderate risk of being diagnosed with diabetes within the next 10 years.

The systematic increase in the prevalence of diabetes, together with the high rate of undiagnosed diabetes and prediabetes [12], indicates the importance of expanding screening for diabetes. Added to that, patients with T2DM may remain asymptomatic and thus go undiagnosed for up to 7 years and many of them, upon finally being diagnosed, present with diabetes-related complications [13]. Furthermore, little is known about the extent of sociodemographic differences and the risk of developing T2DM, primarily due to the paucity of data sources that contain health and sociodemographic information.

Against that background, the objective of our study was twofold: to measure the prevalence of the risk of developing T2DM among adults in Saudi Arabia using a validated risk scoring questionnaire (i.e., ARABRISK) and to evaluate associations between the risk and various sociodemographic variables. Notably, unlike in previous studies, we recruited participants using online snowballing via social media and included all 13 regions of Saudi Arabia.

## 2. Materials and Methods

### 2.1. Study Design and Population

In our cross-sectional survey-based study, conducted in Saudi Arabia between January 2021 and January 2022, we recruited participants using snowballing sampling. The target population was invited to participate by accessing a web link to the survey via ads on social media platforms. To be included in the study, participants had to be between 18 and 74 years old at the time of the survey, be a Saudi national, and be able to read Arabic fluently. All participants diagnosed with diabetes at the time of the survey were excluded from the sample.

### 2.2. Study Questionnaire

In our survey, we used the Arabic version of CANRISK [11], a validated tool with high reliability and validity in Saudi and Jordanian populations that consists of 12 questions used to assess the risk of developing T2DM. Although CANRISK was developed for adults aged 40–74 years old, it can also be used by younger adults, according to the Canadian Task Force on Preventive Health Care’s recommendations on screening for T2DM in adults [14]. A score of ≥43 indicates a very high risk (i.e., 50% probability) of being diagnosed with diabetes within the next 10 years, while a score of 33–42 indicates a high risk (i.e., 33% probability) and a score ≤32 indicates a low to moderate risk (i.e., ≤17% likelihood) [8]. A group of endocrinologists, clinical pharmacists, and researchers evaluated the questionnaire for face validity and determined that the items were clear, comprehensive, and appropriate for the study’s objectives. Additional questions about any diagnoses with T2DM, sociodemographic information (e.g., nationality, region of residence, marital status, and employment status), and type of insurance were added to the survey. The participant’s Internet Protocol (IP) address was automatically retrieved in the survey to confirm their geolocation.

### 2.3. Objectives

The primary objective of our study was to measure the prevalence of the risk of developing T2DM among adults in Saudi Arabia. By extension, the secondary objectives were to analyze the most common risk factors of T2DM in Saudi Arabia, to compare the prevalence of individuals at risk for T2DM between differently sized cities (i.e., large cities, midsize cities, small cities, and rural areas), and to explore the associations between various sociodemographic variables and an increased risk of developing T2DM.

### 2.4. Definitions

We defined a large city as a city with a population of more than 1,000,000, which in Saudi Arabia includes Riyadh, Jeddah, Dammam, Makkah, and Madinah. By contrast, midsize cities had from 300,000 to 1,000,000 people (i.e., Hufuf-Mubarraz, Taif, Tabuk, Buraydah, Jizan, Najran, Albaha, Hail, Jubail, Khamis Mushait, Skaka, and Khobar), whereas small ones had fewer than 300,000 people (i.e., Al Qunfudhah, Ar Rass, Gurayat, Sharurah, Unaizah, Abha, and Yanbu) [15]. All other locations were categorized as rural areas.

Among other definitions, the risk categories were defined according to the original CANRISK questionnaire [8], such that total scores of <32 indicate a low to moderate risk, while scores of ≥33 indicate a high risk.

### 2.5. Sample Size Calculation

Assuming a 20% frequency of individuals at risk of diabetes and the population of Saudi Arabia (i.e., 34,218,169) based on the General Authority for Statistics [15], we calculated a minimum sample size of 385 for our study, with an alpha (α) level of 0.05. However, given the non-probabilistic nondiscriminatory snowball sampling strategy employed, we aimed for a far larger sample in order to potentially compensate for sampling errors and heterogeneity between characteristics of our sample and the general population.

### 2.6. Statistical Analysis

Data were analyzed using R software (R Foundation for Statistical Computing, Version 4.0.1, Vienna, Austria). Descriptive statistics were used for frequencies and percentages of the answers, as well as for the medians and interquartile ranges (IQRs) of total scores. Percentages of the survey categories were compared between the groups of differently sized cities, and the significance of the differences between the groups in relation to the survey categories and ARABRISK total scores were tested with one-way analysis of variance (ANOVA) or χ^2^ for continuous data and Kruskal–Wallis for categorical data. Predictors of high ARABRISK scores were assessed with univariable and multivariable logistic regression models built with sociodemographic variables that were not part of the ARABRISK calculated score—that is, city size, type of insurance, marital status, and employment status. The findings of logistic regression were presented as odds ratios (ORs) with 95% confidence intervals (CIs). Any *p*-value of less than 0.05 was considered to indicate a statistically significant difference between groups of variables included in the logistic regression models.

For the geospatial visualization of our results, we used the R-based package rgeolocate that interfaces with MaxMind GeoLite2 IP databases [16]. Using the ggmap package (Version 3.0.1) to obtain a map of the area as a contextual layer, we were able to map the density of individuals with high ARABRISK scores using the ggplot2 package [17].

## 3. Results

**Figure 1:**
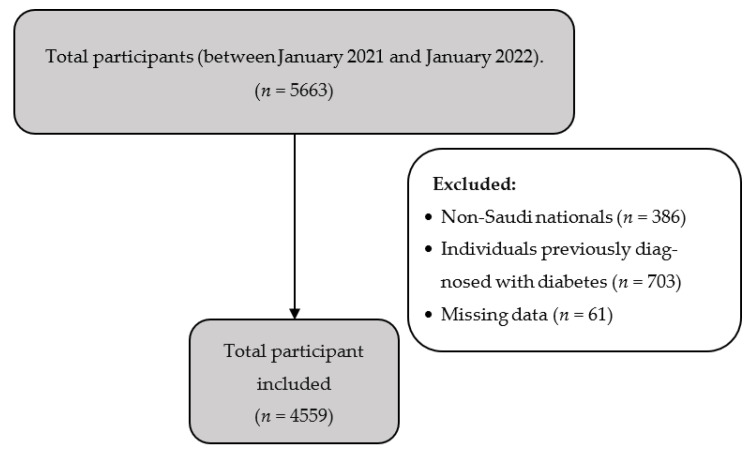
Flowchart of Participants.

Of the 5663 participants who completed the online questionnaire, 4559 participants were included in the analysis, while the rest were excluded for having been diagnosed with diabetes and/or not being Saudi nationals (Figure 1). Approximately half (49.8%, *n* = 2269) of the participants lived in a large city, whereas only 139 (3.0%) lived in a small one. The median calculated ARABRISK score for the whole sample was 16 (IQR = 10 to 25). While 92.5% of the sample had a low to moderate risk of developing T2DM (i.e., <32 points), 7.5% had a high risk (i.e., ≥33 points). The baseline characteristics of the sample and ARABRISK total scores according to groups of differently sized cities are presented in Table 1.

By age, participants ranged from 18 to 74 years old, and 88.1% were between 18 and 39. By sex, three-quarters of the participants were female (74.9%, *n* = 3414), only 4.4% of whom had given birth to a baby weighing 4 kg or more. Approximately half of the participants (48.7%, *n* = 2219) had a body mass index (BMI) of less than 25 kg/m^2^, while 7.8% had a BMI of more than or equal to 35 kg/m^2^. Exercising was reported by 42.1%, while 28.9% reported eating fruits and vegetables on a daily basis. Added to those characteristics, 11.6% of the participants had hypertension, 7.2% had a history of high blood glucose readings, and more than 80% had a family history of diabetes. Last, by level of education, participants were categorized as having completed junior high school or less (2.3%) or having earned a high school degree (30.5%), a college degree (10.3%), or a university degree (56.9%).

**Figure 2:**
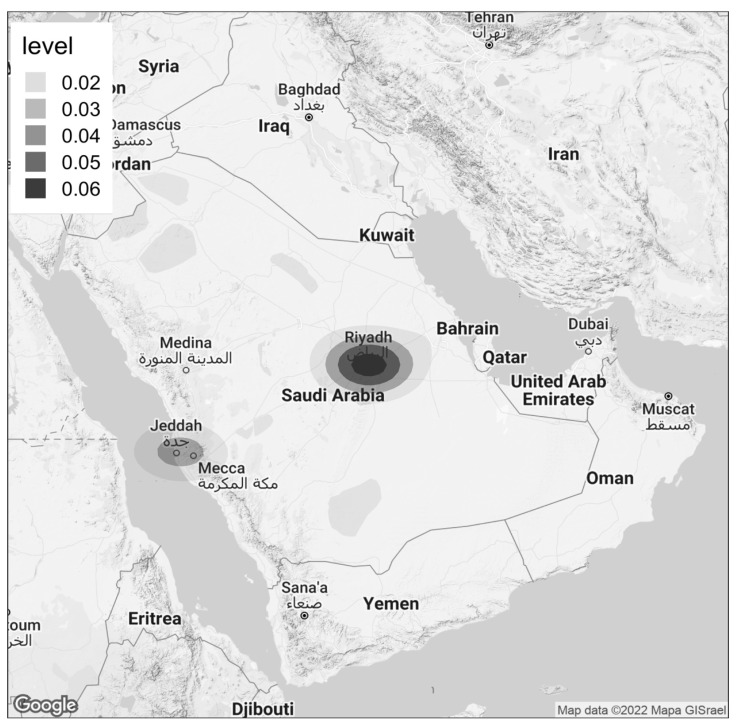
Density of individuals with high ARABRISK score (≥33) using geospatial mapping technique by geolocating IP addresses.

Predictors of the occurrence of high ARABRISK scores were assessed with logistic regression modeling (Table 2). The univariable model revealed that living in a midsize city was associated with a lower ARABRISK score (OR = 0.44, 95% CI = 0.32 to 0.61, *p* < 0.001), as was living in a small city (OR = 0.89, 95% CI = 0.48 to 1.63, *p* = 0.704) or rural area (OR = 0.58, 95% CI = 0.44 to 0.78, *p* < 0.001). However, in the multivariable model, only midsize cities maintained the statistical significance (OR = 0.63, 95% CI = 0.45 to 0.88, *p* = 0.007). Figure 2 shows the density of individuals with a high ARABRISK score (≥33 points) using geospatial mapping by geolocating IP addresses.

Other variables significantly associated with high ARABRISK scores in the univariable model were being married (OR = 6.27, 95% CI = 4.75 to 8.26, *p* < 0.001), divorced or widowed (OR = 6.81, 95% CI = 4.11 to 11.29, *p* < 0.001), or retired (OR = 6.32, 95% CI = 4.20 to 9.50, *p* < 0.001), all of which maintained their statistical significance in the multivariable model. By contrast, the same model revealed that lower ARABRISK scores were associated with having private insurance (OR = 0.67, 95% CI = 0.50 to 0.88, *p* = 0.005) and being unemployed (OR = 0.63, 95% CI = 0.48 to 0.82, *p* < 0.001).

## 4. Discussion

In our web-based, cross-sectional national survey study, we aimed to study the risk of developing T2DM among adult Saudi nationals without diabetes using the ARABRISK questionnaire. In the process, we also investigated the degree to which sociodemographic variables affected the identified level of risk. We believe that the outcomes of our study can be a useful basis for a larger study in the future.

In our study, the prevalence of individuals with high risk of developing T2DM, 7.5%, was less than in previous studies [5,18,19]. The differences could be explained by our mostly younger sample, 88.1% of whom were 18 to 39 years old. The most reported risk factors were a family history of diabetes (81.1%), an inadequate daily intake of fruits and vegetables (71.1%), a lack of exercise (57.9%), and BMI ≥ 25 (51.4%). Residing in a large city, not having private insurance, being retired, and being married, divorced, or widowed were the sociodemographic variables that most predicted high ARABRISK scores.

Major aspects of lifestyle, including frequent car use, low physical activity, obesity, smoking, and diets high in fatty and calorie-dense foods, refined sugar, and salt have been associated with migration into cities [20]. In our study, approximately half of the participants lived in a large city, and they had higher median ARABRISK total scores than participants living elsewhere. That trend is illustrated in the heating map shown in Figure 2, which illustrates the concentration of participants at risk of T2DM in major cities such as Riyadh. All modifiable and non-modifiable T2DM risk factors included in the survey, except a lower degree of education, were more common among participants living in large cities. Unlike our study, a past cross-sectional study among adults with diabetes in Ghana and South Africa revealed that individuals who lived in urban areas were significantly more likely to have the disease [21].

We have also noted high rates of marriage, divorce, widowhood, and retirement among participants living in large cities compared to other cities, all of which were associated with an increased likelihood of high ARABRISK total scores in our multivariable analysis. More than half of participants were single, and only 3.3% were divorced or widowed. Compared with being single, being married, divorced, or widowed increased the risk of T2DM in our analysis.

At the same time, social and cultural aspects also came into play, such that it is difficult to generalize our results to different contexts. Other studies have underscored the importance of marriage in preventing mortality and improving health outcomes [22,23], possibly because married people have partners who consistently support them in maintaining good mental and physical health. Nevertheless, the impact of marital status on having or being at risk of developing T2DM appears to be gender-dependent. In a study examining mortality from diabetes in a large population in Spain, women who were divorced or widowed had the highest mortality of all women, whereas single men had the highest mortality of all men [24]. Meanwhile, also considering the prevalence of T2DM, a study conducted in Iran has revealed that widowed women are less likely to develop T2DM than married women [25].

Following early studies revealing an association between low socioeconomic status (SES) and the prevalence of prediabetes and T2DM [26,27], it is now well known that people who are obese, have a sedentary life style, or have an unhealthy diet are likely to be at higher risk of diabetes and those conditions are more prevalent among individuals with low SES [4,28]. Among adults with diabetes, low SES is associated with many factors known to contribute to adverse health outcomes, including reduced access to and underuse of advised preventive treatment, poor metabolic control, and psychological distress [29]. To that, the results of our multivariable analysis add that unemployed individuals seem to have a significantly lower risk of T2DM than their employed peers. However, a substantial proportion of these were students and, as such, their unemployment does not necessarily reflect their SES.

Beyond that, our analysis revealed that retirement was associated with an increased likelihood of a high ARABRISK score; that outcome can be expected, given that retired individuals tend to be older, which is a known risk factor for T2DM [3]. Last, among our participants, 21.2% had private insurance, which was associated with a decreased risk of high ARABRISK total scores. Not only could lacking private insurance indirectly represent low SES, but private insurance can also be more flexible and accessible than governmental insurance [30]. We thus believe that having private insurance is advantageous, especially due to increasing opportunities for screening and the early detection of diabetes.

Although our results benefit from our study’s large sample size, our findings have several limitations. First, the original CANRISK was validated for adults aged 40–74 years old but can be used among younger adults, according to the Canadian Task Force on Preventive Health Care’s recommendations for screening for T2DM in adults [14]. On that topic, Srugo et al. evaluated the performance of CANRISK among younger adults [31], and their results showed that scores from the original tool performed well among young adults. However, they also suggested lowering the cutoff for high-risk scores from 33 to 19 for young adults to improve the discriminatory power of the tool. In that light, the percentage of participants with a high risk of developing T2DM in our study could be 26% (*n* = 1187). Second, because CANRISK was translated into Arabic and validated by Alghwiri et al. and showed high reliability and validity in Saudi and Jordanian populations [11], we did not conduct a pilot study to validate the survey. Nevertheless, no study to date has confirmed the validity of the tool among young adults in Saudi Arabia. Third, the cross-sectional survey design was liable to have generated some recall or self-report bias. Fourth, we implemented a non-probabilistic, non-discriminatory exponential snowball sampling strategy using social media that provides advantages such as targeting hard-to-reach individuals; however, it may have negatively affected the reliability of our findings because it resulted in the overrepresentation of a sub-population. In our case, most individuals surveyed were female (74.9%), 18 to 39 years old (88.1%), and had a high level of education (67.2%), all of which may limit the generalizability of our findings in the Saudi population.

Fifth, regarding the findings shown in Figure 2, based on geolocating participants at high risk of developing T2DM using their IP addresses, the rgeolocate package that was used depends on the MaxMind database, which is a frequently updated resource that charges fees for more accurate data. Even so, there is no notable difference in the accuracy of the country-level data between the paid and free versions in Saudi Arabia’s case [32]. We also wonder whether the tool’s identification of IP addresses at the city level is in fact accurate [33]. That limitation is due to using Network Address Translation (NAT) and mobile gateway IP addresses, in which case public IP addresses are assigned to many users by service providers. For example, one gateway may host many cities in the same country. In recognizing that limitation, we also note that the data reported by participants themselves aligned with what we gathered using geomapping (Table 2 and Figure 2), which highlighted that large cities tend to house individuals at higher risk of developing T2DM than midsize cities.

In future research conducted to inform public health decision-makers in Saudi Arabia, using a different geospatial mapping technique with Global Positioning System (GPS) data and a mobile application could yield more granular data about the exact location (i.e., within 1 km) of participants [33]. If applied appropriately, then the approach may be used to study interesting patterns within a city, including associations between the presence of health facilities, served and underserved populations, zip codes, green spaces, parks, and other important variables [34,35].

## 5. Conclusions

In our cross-sectional web-based survey study, the risk of developing T2DM was relatively low. Most participants were female, were young, and had a high level of education. The key sociodemographic factors associated with having high ARABRISK scores included living in a large city, being retired, not having private insurance, and being married, divorced, or widowed. At present, Saudi Arabia faces a diabetes epidemic, and against the country’s steadily increased incidence of diabetes in recent years, the widespread use of self-administered risk scores may increase the early detection of prediabetes and diabetes and, in turn, improve its prevention and treatment.

## Figures and Tables

**Table 1:** Baseline characteristics of the surveyed sample based on city size.

Variable	Total (*n* = 4559)	Large City ^†^ (*n* = 2269)	Midsize City ^‡^ (*n* = 1052)	Small City ^§^ (*n* = 139)	Rural Area (*n* = 1099)	*p* Value
Age (years), *n* (%)						<0.001
18 to 39	3653 (88.1)	1680 (74.0)	934 (88.8)	119 (85.6)	920 (83.7)	
40 to 44	380 (8.3)	219 (9.7)	69 (6.6)	8 (5.8)	84 (7.6)	
45 to 54	391 (8.6)	263 (11.6)	42 (4.0)	9 (6.5)	77 (7.0)	
55 to 64	117 (2.6)	94 (4.1)	5 (0.5)	3 (2.2)	15 (1.4)	
65 to 74	18 (0.4)	13 (0.6)	2 (0.2)	0 (0.0)	3 (0.3)	
Female, *n* (%)	3414 (74.9)	1673 (73.7)	833 (79.2)	104 (74.8)	804 (73.2)	0.003
BMI (kg/m^2^), *n* (%)						<0.001
<25	2219 (48.7)	970 (42.8)	562 (53.4)	86 (61.9)	601 (54.7)	
25 to 29	1302 (28.6)	702 (30.9)	276 (26.2)	33 (23.7)	291 (26.5)	
30 to 34	682 (15.0)	392 (17.3)	152 (14.4)	12 (8.6)	126 (11.5)	
≥35	356 (7.8)	205 (9.0)	62 (5.9)	8 (5.8)	81 (7.4)	
Waist circumference (cm), *n* (%)						<0.001
If female						
<80	1824 (40.0)	298 (13.1)	108 (10.3)	22 (15.8)	184 (16.7)	
80 to 88	1239 (27.2)	237 (10.4)	89 (8.5)	12 (8.6)	95 (8.6)	
>88	351 (7.7)	61 (2.7)	22 (2.1)	1 (0.7)	16 (1.5)	
If male						
<94	612 (13.4)	800 (35.3)	482 (45.8)	60 (43.2)	482 (43.9)	
94 to 102	433 (9.5)	661 (29.1)	285 (27.1)	36 (25.9)	257 (23.4)	
>102	100 (2.2)	212 (9.3)	66 (6.3)	8 (5.8)	65 (5.9)	
Exercise activity, *n* (%)	1918 (42.1)	876 (38.6)	478 (45.4)	63 (45.3)	501 (45.6)	<0.001
Daily fruits, *n* (%)	1317 (28.9)	1577 (69.5)	743 (70.6)	111 (79.9)	811 (73.8)	0.007
History of hypertension, *n* (%)	528 (11.6)	275 (12.1)	119 (11.3)	13 (9.4)	121 (11.0)	0.628
History of high blood glucose, *n* (%)	328 (7.2)	182 (8.0)	70 (6.7)	11 (7.9)	65 (5.9)	0.134
History of 4 kg baby delivery, *n* (%)	201 (4.4)	127 (5.6)	30 (2.9)	6 (4.3)	38 (3.5)	<0.001
Positive family history of diabetes, *n* (%)						
Mother	1235 (27.1)	689 (30.4)	242 (23.0)	34 (24.5)	270 (24.6)	<0.001
Father	1664 (36.5)	898 (39.6)	318 (30.2)	54 (38.8)	394 (35.9)	<0.001
Siblings	742 (16.3)	422 (18.6)	134 (12.7)	23 (16.5)	163 (14.8)	<0.001
Sons	55 (1.2)	31 (1.4)	8 (0.8)	1 (0.7)	15 (1.4)	0.432
Education, *n* (%)						<0.001
Junior high school or less	105 (2.3)	63 (2.8)	18 (1.7)	4 (2.9)	20 (1.8)	
High school	1389 (30.5)	604 (26.6)	368 (35.0)	51 (36.7)	366 (33.3)	
College	471 (10.3)	224 (9.9)	122 (11.6)	10 (7.2)	115 (10.5)	
University	2594 (56.9)	1378 (60.7)	544 (51.7)	74 (53.2)	598 (54.4)	
Insurance *, *n* (%)						<0.001
Private	967 (21.2)	626 (27.6)	180 (17.1)	20 (14.4)	141 (12.8)	
Government	3592 (78.90)	589 (72.4)	872 (82.9)	119 (85.6)	859 (87.2)	
Marital status *, *n* (%)						<0.001
Single	2626 (57.6)	1114 (49.1)	701 (66.6)	100 (71.9)	711 (64.7)	
Married	1781 (39.1)	1063 (46.8)	323 (30.7)	34 (24.5)	361 (32.8)	
Divorced or widowed	152 (3.3)	92 (4.1)	28 (2.7)	5 (3.6)	27 (2.5)	
Employment *, *n* (%)						<0.001
Employed/Self-employed	1309 (28.7)	770 (33.9)	223 (21.2)	28 (20.1)	288 (26.2)	
Unemployed	3138 (68.8)	1414 (62.3)	820 (77.9)	108 (77.7)	796 (72.4)	
Retired	112 (2.5)	85 (3.7)	9 (0.9)	3 (2.2)	15 (1.4)	
ARABRISK score category, *n* (%)						<0.001
Low to moderate risk (<32 score)	4218 (92.5)	2051 (90.4)	1005 (95.5)	127 (91.4)	1035 (94.2)	
High risk (≥33)	341 (7.5)	218 (9.6)	47 (4.5)	12 (8.6)	64 (5.6)	
Calculated ARABRISK score, median (IQR) *	16 (10 to 25)	16 (10 to 25)	13 (8 to 28)	12 (8 to 22)	13 (8 to 21)	<0.001

* These variables are not part of the calculated scores. ^†^ Large city (>1,000,000 population): Riyadh, Jeddah, Dammam, Makkah, and Madinah. ^‡^ Midsize city (300,000 to 1,000,000 population): Hufuf-Mubarraz, Taif, Tabuk, Buraydah, Jizan, Najran, Albaha, Hail, Jubail, Khamis Mushait, Skaka, and Khobar. ^§^ Small city (<300,000 population): Al Qunfudhah, Ar Rass, Gurayat, Sharurah, Unaizah, Abha, and Yanbu. ^¶^ Statistical test between large, midsize, small cities, and rural areas. Abbreviations: BMI, body mass index; IQR: interquartile range.

**Table 2:** Univariable and multivariable logistic regression models to explain high ARABRISK score among the surveyed sample.

Model Type	Univariable Model Odds Ratio (95% Confidence Interval, *p* Value)	Multivariable Model Odds Ratio (95% Confidence Interval, *p* Value)
Variable		
City size		
Large city ^†^	Reference	Reference
Midsize city ^‡^	0.44 (0.32 to 0.61, *p* < 0.001)	0.63 (0.45 to 0.88, *p* = 0.007)
Small city ^§^	0.89 (0.48 to 1.63, *p* = 0.704)	1.31 (0.69 to 2.51, *p* = 0.408)
Rural area	0.58 (0.44 to 0.78, *p* < 0.001)	0.74 (0.55 to 1.00, *p* = 0.502)
Type of insurance		
Government	Reference	Reference
Private	1.03 (0.79 to 1.35, *p* = 0.818)	0.67 (0.50 to 0.88, *p* = 0.005)
Marital status		
Single	Reference	Reference
Married	6.27 (4.75 to 8.26, *p* < 0.001)	4.42 (3.24 to 6.03, *p* < 0.001)
Divorced or widowed	6.81 (4.11 to 11.29, *p* < 0.001)	4.40 (2.57 to 7.54, *p* < 0.001)
Employment status		
Employed	Reference	Reference
Unemployed	0.34 (0.27 to 0.44, *p* < 0.001)	0.63 (0.48 to 0.82, *p* < 0.001)
Retired	6.32 (4.20 to 9.50, *p* < 0.001)	4.63 (3.06 to 7.00, *p* < 0.001)

Binary outcome (low to moderate versus high scores). ^†^ Large city (>1,000,000 population): Riyadh, Jeddah, Dammam, Makkah, and Madinah. ^‡^ Midsize city (300,000 to 1,000,000 population): Hufuf-Mubarraz, Taif, Tabuk, Buraydah, Jizan, Najran, Albaha, Hail, Jubail, Khamis Mushait, Skaka, and Khobar. ^§^ Small city (<300,000 population): Al Qunfudhah, Ar Rass, Gurayat, Sharurah, Unaizah, Abha, and Yanbu.

## Data Availability

The data collected and analyzed during the study are available from the corresponding authors upon reasonable request.
